# The Dual Effect of Selenium Application in Reducing Fusarium Wilt Disease Incidence in Banana and Producing Se-Enriched Fruits

**DOI:** 10.3390/plants13233435

**Published:** 2024-12-06

**Authors:** Lina Liu, Chengye Wang, Kesuo Yin, Ming Ni, Yue Ding, Chengyun Li, Si-Jun Zheng

**Affiliations:** 1 Yunnan Key Laboratory of Green Prevention and Control of Agricultural Transboundary Pests, The Ministry of Agriculture and Rural Affairs International Joint Research Center for Agriculture, Agricultural Environment and Resource Research Institute, Yunnan Academy of Agricultural Sciences, Kunming 650205, China; handyliu@126.com (L.L.); yinkesuo@163.com (K.Y.); ynnkynm1980@126.com (M.N.); dingyue9797@163.com (Y.D.); 2 State Key Laboratory for Conservation and Utilization of Bio-Resources in Yunnan, Yunnan Agricultural University, Kunming 650500, China; 3 Institute of Highland Forest Science, Chinese Academy of Forestry, Kunming 650224, China; cywang11@126.com; 4 Bioversity International, Kunming 650205, China

**Keywords:** banana, *Fusarium oxysporum* f. sp. *cubense* tropical race 4, Se-enriched fruits, selenium, antioxidant enzymes

## Abstract

Fusarium wilt disease severely constrains the global banana industry. The highly destructive disease is caused by *Fusarium oxysporum* f. sp. *cubense*, especially its virulent tropical race 4 (*Foc* TR4). Selenium (Se), a non-essential mineral nutrient in higher plants, is known to enhance plant resistance against several fungal pathogens. The experiments we conducted showed that selenium (≥10 mg/L) dramatically inhibited the growth of *Foc* TR4 mycelia and promoted plant growth. The further study we performed recorded a substantial reduction in the disease index (DI) of banana plants suffering from *Foc* TR4 when treated with selenium. The selenium treatments (20~160 mg/L) demonstrated significant control levels, with recorded symptom reductions ranging from 42.4% to 65.7% in both greenhouse and field trials. The DI was significantly negatively correlated with the total selenium content (TSe) in roots. Furthermore, selenium treatments enhanced the antioxidant enzyme activities of peroxidase (POD), polyphenol oxidase (PPO), and glutathione peroxidase (GSH-Px) in banana. After two applications of selenium (100 and 200 mg/plant) in the field, the TSe in banana pulps increased 23.7 to 25.9-fold and achieved the Se enrichment standard for food. The results demonstrate that selenium applications can safely augment root TSe levels, both reducing Fusarium wilt disease incidence and producing Se-enriched banana fruits. For the first time, this study has revealed that selenium can significantly reduce the damage caused by soil-borne pathogens in banana by increasing the activities of antioxidant enzymes and inhibiting fungal growth.

## 1. Introduction

Banana (*Musa* spp.) is extensively cultivated in 135 countries, primarily in tropical and subtropical regions across the globe. It is a vital food and economic crop that serves as a staple for more than 600 million people [1]. Banana is the most widely exported fresh fruit, valued at around USD 10 billion per year. The global banana trade has recorded approximately 20 million tons annually in recent years (Available online: www.fao.org). Cavendish cultivars constitute most bananas cultivated for export. They belong to the triploid variety of “dessert” banana derived from *M. acuminata*. Unfortunately, the sustainable development of the banana industry is seriously threatened by Fusarium wilt, a disease caused by *Fusarium oxysporum* f. sp. *cubense* (*Foc*) fungus, where Tropical Race 4 (TR4) is particularly virulent and destructive. *Foc* TR4 infects various banana plant tissues, including root, corm, pseudostem, leaf, and peduncle tissues [2]. In recent decades, the pathogen has spread rapidly, and to date, the disease has extended beyond Asia, affecting over 20 countries in the EPPO region, Africa, South America and Oceania, including Colombia, Jordan, Mozambique, Oman, Peru, Australia, and Grande Comoros [3,4,5,6,7,8,9]. Control efforts against *Foc* TR4 have been immensely challenging, since the disease cannot be adequately managed with fungicides, soil fumigants, or conventional cultivation practices such as crop rotation and soil amendment [6,10]. Due to *Foc* TR4’s rapid spread and difficulty to control, many banana plantations in China have been devastated and abandoned [11,12]. The most effective measure for controlling the disease is breeding disease-resistant varieties. For example, replacing susceptible cultivar Gros Michel with the Cavendish cultivar is a classic successful case in *Foc* race 1 disease control [5]. However, a cultivar of banana resistant to wilt disease caused by *Foc* TR4 has not yet been created. It is imperative to search for a range of alternative *Foc* TR4 control solutions.

Selenium (Se) is a beneficial element for both animals and plants. It was first discovered as a toxic element by Swedish chemist Berzelius in 1817 and was not confirmed as an essential trace element for animals until 1957 [13]. It was found to be a necessary component of antioxidant enzyme glutathione peroxidase in rats [14]. Most of China’s land is Se-deficient [15]. Se intake is closely related to human health and plays important roles in anti-aging, anti-tumor, and disease prevention mechanisms [16,17]. Se intake in the human body is mainly determined by Se content in food, so the conversion of Se in animals and plants has also received significant attention. Extensive research has been conducted on Se enrichment in crops with the aim of improving crop quality and yield [17,18,19,20,21,22]. In banana, it has been found that spraying Se-containing fertilizer on leaves and applying sodium selenate (Na_2_SeO_4_) solution to the soil can increase both banana yield and Se content [23,24]. Se treatments during the banana plantlet stage can enhance levels of soluble sugars, potassium, vitamin C, and Se in banana fruit and reduce toxic malondialdehyde content in banana leaves [24,25].

Se can mediate plant resistance against pathogenic fungi by preventing their invasion and inhibiting their growth [21,26,27,28,29]. Hanson et al. (2003) [26] discovered that Se could exert both direct and indirect effect against the pathogens (*Fusarium* sp.) in Chinese Mustard (*Brassica juncea*), such as by inhibiting the growth of pathogens and reducing the adverse impact on seedling growth when inoculated with the pathogens. Se exhibited a strong inhibitory effect on the incidence of Sclerotinia stem rot in oilseed rape, as it altered the structure and function of the soil microbial community, which helped prevent pathogen invasion [27]. Se can directly inhibit mycelia growth, spore germination, and germ tube elongation and disrupt the membrane integrity of fungal pathogens, thereby distorting/deforming the fungal structure and decreasing the accumulation of fungal toxins (aflatoxin and deoxynivalenol) [30,31,32,33,34,35]. Se also stimulates plants develop important defense mechanisms against pathogens, including by activating defense genes or producing secondary metabolites to mediate host immunity and signal transduction, thereby resisting fungal pathogens [29]. Se application was found to significantly increase levels of aspartic acid, glutamine, L-glutamic acid, tryptophan, and tyrosine in plants after inoculation with *Sclerotinia sclerotiorum* [36], which have been identified as resistance-related metabolites against pathogens [37,38,39]. Se applications increase its bioconcentration in tissues, as well as the activity of antioxidant enzymes such as catalase, polyphenol oxidase, and peroxidase in plants, to better resist pathogens [27,36,40,41]. In addition, Se leads to the upregulation of defense genes, including *CHI*, *ESD1*, *NPR1*, and *PDF1.2*, in plant leaves [36]. Thus, it can be concluded from the above studies that utilizing Se to control disease could offer a novel and effective solution. After infection with *Foc* TR4, bananas accumulate H_2_O_2_, which, in turn, triggers the production of reactive oxygen species (ROS) [42,43]. Se plays a crucial role in modulating the enzymatic activities of the antioxidant system, thereby facilitating the maintenance of ROS homeostasis and improving disease resistance in tomato [40,42,44]. However, to date, no reports on Se’s ability, concentrations, or application methods to mitigate *Foc* TR4 infection in banana are available.

To explore the effects of Se treatments on Fusarium wilt *Foc* TR4, one popular banana variety, the Brazilian variety (*Musa* spp., Cavendish subgroup, AAA, susceptible), was used in this study. We investigated the effect of Se application on the growth of banana plantlets in a greenhouse and *Foc* TR4 mycelia in vitro, as well as the reduction in Fusarium wilt caused by *Foc* TR4 and the Se enrichment of different banana plant tissues, with particular focus on the edible part (banana fruits). The objective of this study is to evaluate if there is a positive regulatory role of Se in reducing *Foc* TR4 incidence and provide an alternative option for the development of a sustainable *Foc* TR4 disease management strategy in the field, producing Se-enriched banana at the same time.

## 2. Results

### 2.1. The Effect of Se Treatments on Banana Plantlet Growth in a Greenhouse

**Figure 1:**
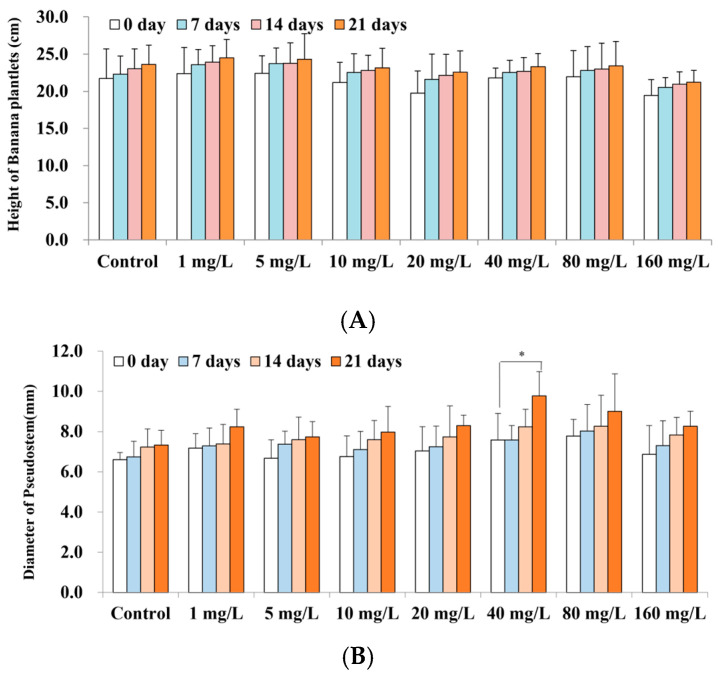

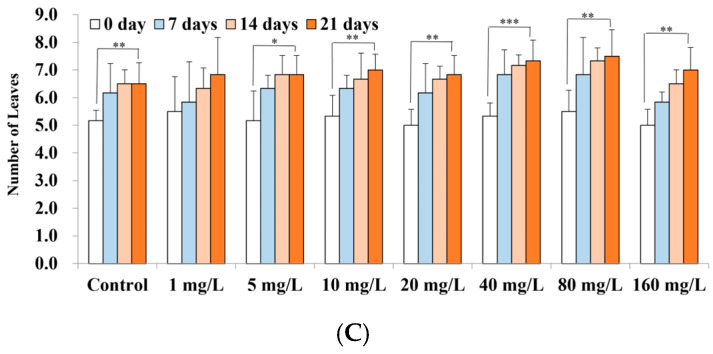
The effect of Se on banana plantlet growth. The graphs illustrate the effects on different concentrations of Se on banana plant height (**A**), the diameter of the pseudostem (**B**), and the number of leaves (**C**). Note: The data are presented as the mean ± Standard Error (SE), with *n* = 6 replicates. Statistical significance was determined between the treatment and the control using Student’s *t*-tests, with * *p* < 0.05, ** *p* < 0.01, and *** *p* < 0.001.

The effects of varying concentrations of Se on the growth of banana plantlets are shown in Figure 1. The graphs illustrate the effects of different concentrations of Se on banana plant height (A), the diameter of the pseudostem (B), and the number of leaves (C). The data show that the height, the diameter of the pseudostem, and the number of leaves of banana plantlets treated with Se exhibited an increase over time, surpassing the initial baseline of 0 days. Compared to the control, the height increments were more pronounced in the 1–20 mg/L Se treatments, whereas at concentrations above 40 mg/L, growth was reduced, suggesting that low Se concentrations stimulated growth, while high concentrations hindered it, although the effect was not statistically significant. The diameter of the pseudostem increased from 1.07 cm to 2.21 cm after 21 days of treatments with various Se concentrations, with the greatest increase observed at 40 mg/L, corresponding to a significant increase of 2.0 times relative to the control (0.73 cm) (*p* < 0.05). The number of leaves also increased by 1.3 to 2.0, and the changes in leaf number for treatments with Se concentrations greater than 5 mg/L were statistically significant (*p* < 0.05) compared to the control (1.3 leaves). Overall, the data demonstrate that Se treatments (≥5 mg/L) enhanced the diameter of the pseudostem and promoted leaf growth in banana plantlets.

### 2.2. The Foc TR4 Mycelia Growth-Controlling Effect of Se Treatments In Vitro

**Figure 2:**
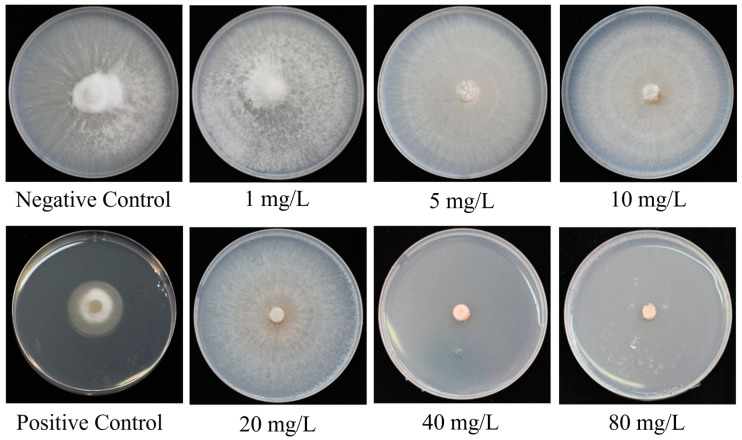
The effect of Se on the in vitro growth of *Foc* TR4 mycelia at 7 dpi. The graphs illustrate the colony diameter of *Foc* TR4 on PDA medium containing Se. Note: The negative control was PDA medium without Se. The positive control was PDA medium with 30% pyrazole ether fungicide suspension (10 mL/L), which is a broad-spectrum fungicide.

The in vitro growth of *Foc* TR4 mycelia with varying concentrations of Se is shown in Table 1 and Figure 2. The data demonstrate that the colony diameter of *Foc* TR4 on PDA medium was significantly inhibited compared to the control within the first 3 days of treatment at concentrations of 20 mg/L and higher (*p* < 0.05). Additionally, concentrations of 5 mg/L and higher for more than 4 days exhibited a significant inhibitory effect on the growth of *Foc* TR4 mycelia, with the inhibitory effect being extremely significant at concentrations of 40 mg/L and higher (*p* < 0.001). On the 6th day of cultivation, the inhibition rates of Se on PDA medium were 8.2%, 16.8%, 93.0%, 97.1%, and 97.7% at concentrations of 10 mg/L and higher. These findings suggest that Se exerted a pronounced inhibitory effect on the growth of *Foc* TR4 in vitro.

### 2.3. The Foc TR4-Controlling Effect of Se Treatments in the Greenhouse and Field

**Figure 3:**
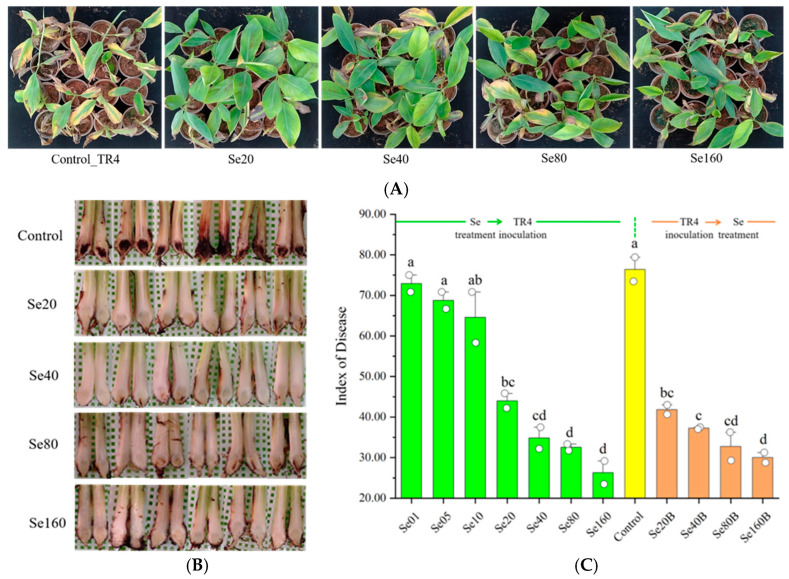
The effect of Se in reducing *Foc* TR4 incidence in banana plants in greenhouse experiments. (**A**) Different above-ground symptoms of banana plants under Se treatments and inoculated with *Foc* TR4. Only images of plants treated with Se that exhibited significant reductions in DI with respect to the control are shown. (**B**) Different corm symptoms in banana plants under Se treatments and inoculated with *Foc* TR4. (**C**) The change in the *Foc* TR4 disease index of banana plants after treatment with different concentrations of Se. The data represent the mean ± SE, with 10 plants as replicates. Note: Se01–Se160 denote the Se treatment concentrations prior to *Foc* TR4 inoculation, whereas Se20B–Se160B signify the Se treatment concentrations post *Foc* TR4 inoculation. The letters a–d represent statistical significance determined using Student’s *t*-tests, and the absence of shared letters signifies a significant difference at the *p* < 0.05 level.

To demonstrate the *Foc* TR4-controlling effect of SE treatment on banana plantlets, we conducted assessments of disease resistance using two in-greenhouse methods. The experimental results show that before *Foc* TR inoculation, Se-treated plantlets showed above-ground and underground wilting symptoms, which were alleviated after treatment with Se. The average DI values were 72.92, 68.75, 64.58, 44.01, 34.84, 32.54, and 26.30 with the corresponding increases in Se concentration. The DIs of plantlets treated with more than 20 ppm Se were significantly lower than that of the control (DI = 76.42), resulting in a disease control efficacy of 42.4% to 65.7% (Figure 3A–C, Se01–Se160). On the other hand, the results of the experiment with plantlets inoculated against *Foc* TR4 before they were treated with Se were consistent with plantlets first Se-treated, then *Foc* TR4-inoculated (Figure 3C, Se20B–Se160B, Figure S1). The data demonstrate that plantlets’ DIs significantly decreased, ranging from 30.00 to 41.82, resulting in a disease control efficacy ranging from 45.3% to 60.6%. At Se concentrations of 20 mg/L and greater, DI was not significantly different any more between Se treatments before and after inoculation with *Foc* TR4 for each Se concentration. These results suggest that banana plantlets that received Se treatments (more than or equal to 20 mg/L) demonstrated a significant *Foc* TR4-controlling effect.

**Figure 4:**
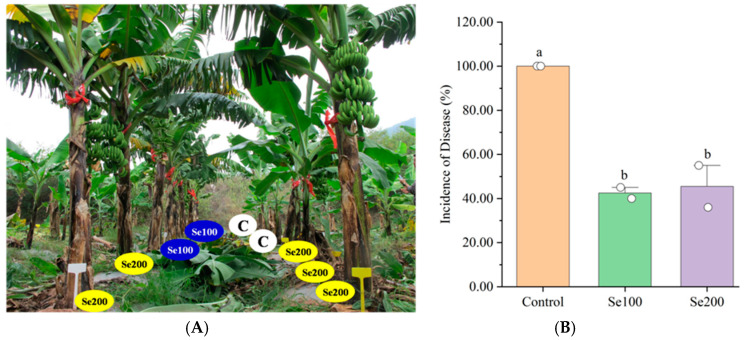
The effect of Se in reducing *Foc* TR4 incidence in banana plants in field experiments. (**A**) The effect of Se treatments in the field in reducing *Foc* TR4 incidence in banana plants during the fruiting stage. (**B**) The change in the *Foc* TR4 disease index of banana plants treated with different doses of Se. The data represent the mean ± SE, with 10 plants as replicates. The letters a, b represent the statistical significance determined using Student’s *t*-tests, and the absence of shared letters signifies a significant difference at the *p* < 0.05 level. Note: In (**A**), the plants labeled as C on the right of the banana plants treated with Se are the new suckers that emerged after the death of non-fruiting plants due to *Foc* TR4 infection.

This research also investigated the impact of Se treatments on *Foc* TR4 in the field. A banana plantation displaying moderate *Foc* TR4 levels was selected, where banana plants suffered from severe wilt disease in the later stages (Figure 4 and Figure S2). Banana plants showing no disease symptoms during vegetative growth were treated with Se100 and Se200 at levels of 100 and 200 mg/plant, respectively (Figure 4A). The Se-treated banana plants exhibited significantly stronger resistance to *Foc* TR4, and banana fruits were successfully harvested (Figure 4B). Data analysis revealed that the average disease incidence of banana plants was 42.50 and 45.5 under Se100 and Se200 treatments, respectively, significantly lower than the control group (disease incidence = 100). These results suggest that Se significantly reduced the detrimental effect of *Foc* TR4 on banana plants in the field.

### 2.4. TSe in Roots Is Negatively Correlated with the Disease Index Under Se Treatments

**Figure 5:**
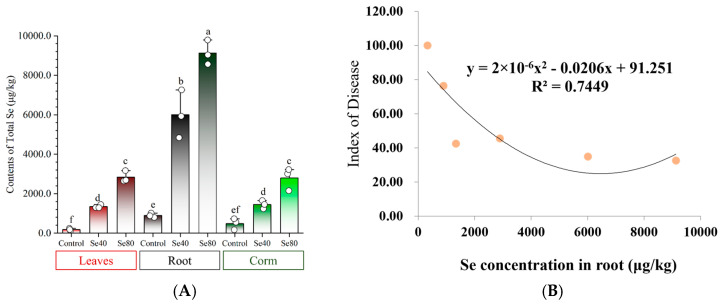
Changes in total Se content in different banana tissues after Se application. (**A**) The total Se content in different tissues of banana plantlets after Se application in greenhouse experiments. Data are presented as the mean ± SE, with *n* = 3 replicates. The letters a–f represent the statistical significance determined using Student’s *t*-tests, and the absence of shared letters signifies a significant difference at the *p* < 0.05 level. (**B**) Curvilinear correlation between DI and Se concentration in roots.

To verify the absorption of Se by banana plants, we measured the TSe with different concentrations and tissues in greenhouse-grown plantlets and field-grown mature plants (Figure 5A, Table 2). In the greenhouse experiments, the concentrations of TSe were significantly higher in the roots (4839~9791 μg/kg) compared to the leaves (1292~3166 μg/kg) and corms (1234~3218 μg/kg) (Figure 5A). Root TSe was approximately four times higher than that in the leaves and corms. Leaf TSe in Se80, corm TSe in Se80, leaf TSe in Se40, and corm TSe in Se40 were 2831.3 ± 237.0, 2792.0 ± 463.8, 1343.0 ± 75.0, and 1446.7 ± 170.7 μg/kg, respectively, showing significant variation between the two Se treatment concentrations. Additionally, no notable difference was found between the leaf and corm under the same Se treatment concentration (Figure 5A). However, for the Se treatments conducted in the field, TSe levels were highest in Root_Se200 (2891.0 ± 770.9 μg/kg), followed by Root_Se100 (1337.7 ± 88.9 μg/kg), then Peduncle_Se200, Peduncle_Se100, Pseu_Se200, Pseu_Se100, Leaf_Se200, Leaf_Se100, Corm_Se200, and Corm_Se100 (488.7 ± 321.1~128.7 ± 28.2 μg/kg) (Table 2). The TSe in different banana tissues followed this order: root > peduncle = pseudostem ≥ leaf = corm. Furthermore, significant variations in TSe between the Se100 and Se200 treatments were observed in the root and corm. Similarly, there was no significant difference in TSe between the leaf and corm under the same treatment dosage. These results reveal that the TSe in plant tissues is mainly influenced by the Se treatment level, with the root as the primary tissue for Se accumulation in banana plants.

To assess the correlation between DI and TSe in tissues, the DIs of banana plantlets under Se treatments applied in the greenhouse and in the field, as well as the TSe in all tested tissues, were used for analysis. The result revealed that the DI had the most significant negative correlation with the TSe in the root (R = −0.86, *p* < 0.01) (Figure 5B), while the DI had no significant negative correlation with TSe in leaf (R = −0.72) or corm (R = −0.59). These results indicate that *Foc* TR4 incidence was reduced due to banana roots containing Se, where higher Se doses are more effective.

### 2.5. Se Effectively Alleviates Foc TR4 Incidence to Successfully Produce Fruits Within the Safe Threshold

**Figure 6:**
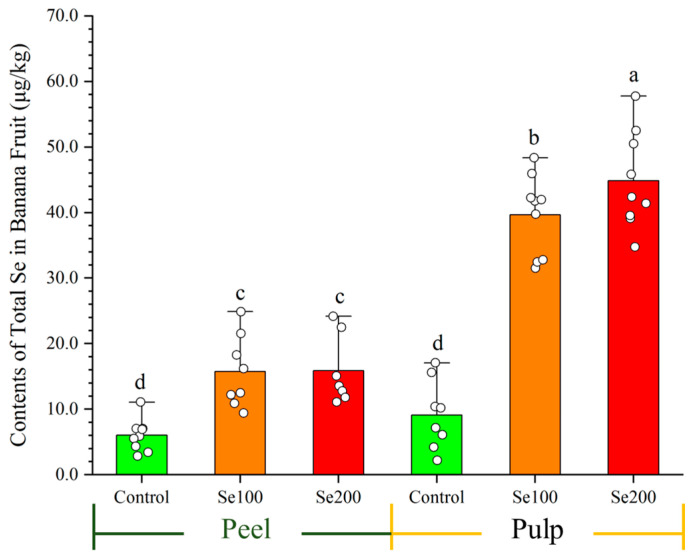
The total Se content of fresh banana (peel and pulp) after Se application. Data are presented as the mean ± SE, with *n* = 9 replicates. The letters a–d represent the statistical significance determined using Student’s *t*-tests, and the absence of shared letters signifies a significant difference at the *p* < 0.05 level.

To ensure fruit safety, we determined the TSe in the peel and pulp of green banana (Figure 6). The data show that the Se100 and Se200 treatments resulted in significantly higher Tse contents in pulp and peel compared to the control. Moreover, the Tse levels exhibited a significant disparity, with a higher concentration observed in the pulp compared with the peel, suggesting that banana pulp has a greater tendency to accumulate Se. The corresponding pulp Tse contents were 39.6 ± 5.7 μg/kg and 43.2 ± 5.6 μg/kg, increasing by 23.7 fold and 25.9 fold, respectively, over the control (1.7 ± 0.9 μg/kg); these Tse levels are within the standard for Se-enriched fruit (10–100 μg/kg, DB45/T1061-2014 [45]). These results illustrate that Se treatments within the safety threshold can effectively achieve control of *Foc* TR4 in banana plants while also producing Se-enriched fruits.

### 2.6. Se Triggers Indicators of the Antioxidant System in Banana to Enhance Resistance Against Foc TR4

**Figure 7:**
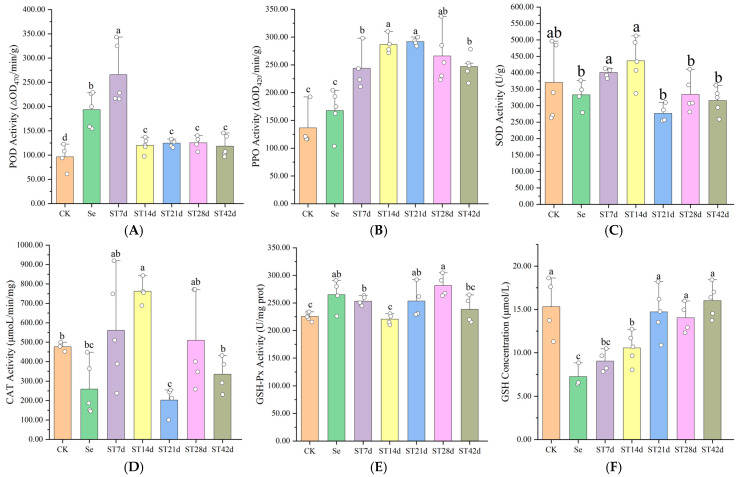
Changes in antioxidant system indicators of eliminating ROS after Se and TR4 treatments, as well as POD activity (**A**), PPO activity (**B**), SOD activity (**C**), CAT activity (**D**), GSH-Px activity (**E**), and GSH concentration (**F**). Data are presented as the mean ± SE, with *n* = 5 replicates. The letters a–c represent the statistical significance determined using Student’s *t*-tests, and the absence of shared letters signifies a significant difference at the *p* < 0.05 level. Note: CK: control; Se: banana leaves treated with Se for 21 d; ST7d: banana leaves treated with Se for 21 d and *Foc* TR4 for 7 d; ST14d: banana leaves treated with Se for 28 d and *Foc* TR4 for 14 d; ST21d: banana leaves treated with Se for 35 d and *Foc* TR4 for 21 d; ST28d: banana leaves treated with Se for 42 d and *Foc* TR4 for 28 d; ST42d: banana leaves treated with Se for 56 d and *Foc* TR4 for 42 d. The concentration of Se treatment was 40 mg/L.

To further explore the relationship between Se and banana resistance, we quantified the Se content in leaves at various stages and assessed indicators of the antioxidant system, including the enzyme activities of peroxidase (POD), polyphenol oxidase (PPO), superoxide dismutase (SOD), catalase (CAT), and glutathione peroxidase (GSH-Px), as well as the reduced glutathione (GSH) concentration. The detailed data are presented in Figure 7. The data reveal that the highest Se content in leaves was 440.0 ± 50.6 μg/kg in the ST7d treatment, which is significantly higher than the levels of 182.0 ± 28.5 μg/kg in ST21d and 140.0 ± 24.8 μg/kg in ST42d (*p* < 0.01). The control treatment exhibited a Se content of 14.0 ± 3.0 μg/kg (*p* < 0.001). These data suggest that as banana plants grow further, the Se content in plant tissues diminishes. Following Se treatment, both POD and PPO activities increased, with a more pronounced rise observed after inoculation with *Foc* TR4, with the highest increases of 2.76 times and 1.78 times (*p* < 0.01), respectively. As the Se concentration decreased, POD activity declined, while PPO activity remained high, with a 2.13 times increase in ST21d (*p* < 0.001). No correlation was observed between the SOD or CAT activities and treatment. The activity of GSH-Px significantly increased by 12.3% to 24.8%, whereas the GSH concentration significantly decreased by 31.1% to 52.5% post Se treatment (*p* < 0.05). As the Se content decreased, the GSH concentration returned to normal levels. The results indicate that POD, PPO, and GSH-Px play a role in enhancing the antioxidant response in bananas and contribute to the resistance to TR4 induced by Se.

## 3. Discussion

### 3.1. Se Diminishes Foc TR4 Damage and Promotes Banana Growth

So far, there are no commercially available banana varieties that are immune to *Foc* TR4 [6,12]. Thus, the *Foc* TR4 susceptibility of the widely cultivated Brazilian variety severely restricts the sustainable development of the banana industry. Moreover, there is a lack of effective chemical agents for preventing and controlling *Foc* TR4, which means that heavily infected banana plantations can only be abandoned or rotated with other crops. In this study, we reported, for the first time, that a form of Se (Se^4+^) applied using the recommended dosage can reduce the detrimental effects of *Foc* TR4 on banana plants. After banana plantlets were treated with a specific dose of Se in the greenhouse, we observed a control effect of over 60% against *Foc* TR4. The control effect of banana plants grown in a field treated with Se (100 mg/plant) reached up to 57%, allowing a successful fruit harvest.

Some studies have demonstrated Se’s potential for reducing some harmful effects of pathogens. For instance, Se has been shown to effectively reduce fungal diseases caused by *Fusarium* spp. and *S. sclerotiorum* in Brassicaceae plants (*B. juncea* and *B. napus*), in Solanaceae plants (*Solanum lycopersicum*) and in Helianthus plants (*Helianthus annuus*) [26,27,28,40,46]. Research has also indicated that Se can reduce citrus Huanglongbing caused by the *Candidatus* Liberibacter asiaticus bacterium [47]. Our study has proven that Se application can control Fusarium *Foc* TR4 in banana. Previous studies included dicotyledonous host crop plants of citrus, rapeseed, mustard, and tomato, belonging to the Rutaceae, Cruciferae, and Solanaceae families, respectively. However, banana belongs to the monocotyledonous Musaceae family, with more developed adventitious roots that can grow up to 100–300 cm in breadth and penetrate the soil up to 120–150 cm in depth. Our findings show that Se can significantly reduce the damage caused by soil-borne diseases in monocotyledonous plants with well-developed root systems.

Moreover, Se treatments have been proven to promote the diameter of the pseudostem and increase the height of banana plants [29]. Our research found that Se has a promoting effect on the growth of Banana plantlets, including the diameter of the pseudostem and the leaf number. This effect was influenced by the concentration of Se. We found that low concentrations of Se treatment in this study promoted an increase in plant height, while high concentrations had a negative effect. However, the differences were not significant. The discrepancies in the outcomes can be attributed to the differences in the types of Se compounds, dosage levels, and plant varieties [29,48,49,50]. Our research has demonstrated that Se^4+^ (≤40 mg/L) exhibited a beneficial effect on the growth of Brazilian plantlets, which are cultivated widely in banana production. Se is not an essential element for plants, yet an appropriate dose can promote plant growth. However, elevated levels of Se can inhibit growth and may even prove toxic [51]. The factors of plant species, Se form, concentration, and plant status are pertinent to these effects [50,51]. Banana is a non-Se accumulator with a relatively narrow tolerance range for Se. Our findings indicate that concentrations of 5–40 mg/L Se^4+^ have a promoting effect on banana plantlets in the greenhouse, laying the foundation for the potential utilization of Se in banana cultivation. However, there are notable differences in the utilization of Se among various banana varieties, growth environments (plantlets grown in a greenhouse versus tissue cultured in bottles), and forms of Se within tissues (personal observation). How Se enhances growth promotion in banana requires further study.

### 3.2. The Se Form Reducing the Disease Index Is Correlated with TSe in Banana Roots

Se has proven to be effective in controlling fungal pathogens, but the specific chemical form is important [29]. Se is a non-essential mineral nutrient in plants, serving as the active center of glutathione peroxidase (GSH-Px) to eliminate harmful peroxides. Two main forms of Se (Se^6+^ and Se^4+^) exist in soil, accumulating in various tissues to different extents. While Se^6+^ rapidly transfers to stems, leaves, and other parts via the roots, Se^4+^ transforms into organic Se compounds (SeMet and SeMseCys) present in the roots [52,53,54,55]. *Foc* TR4 infiltrates banana plants via their roots, thereby establishing the roots as the primary defense barrier against *Foc* TR4. The choice of Se^4+^ as an exogenous treatment placed near banana corm facilitates the accumulation of sufficient Se in roots. The TSe in plant tissue is influenced by the applied Se dose, and it was found that Na_2_SeO_3_ is concentrated mainly in roots [24]. In our study, the TSe in roots reached up to 9.13 mg/kg, which is four times higher than the TSe in other banana tissues. In the fruit-setting stage of bananas in the field, the TSe can reach 2.89 mg/kg in the roots, which 3.9 to 18.8 times higher than in other tissues. The results from this study show a significant negative correlation between the TSe in roots and the DI of plants. The results are consistent with a negative correlation between the incidence rate of Sclerotinia stem rot and plant Se concentration in brassicas [27], but we found that the TSe in banana roots is crucial for alleviating the damage caused by *Foc* TR4 but not in the corm, leaf, pseudostem, or peduncle. A certain dose of Se could enhance plant resistance to pathogen invasion, which may also serve as an inherent driving force for plants to take up Se [51]. Thus, enriching the roots of banana plants with Se can stimulate the disease response, thereby limiting the invasion of *Foc* TR4, which could be an important route for Se to trigger plant resistance.

### 3.3. The Defensing Mechanisms of Se Enhance Banana Resistance Against Foc TR4

Se enhances plant resistance to diseases by inhibiting fungal growth and regulating enzymes in the antioxidant system. The data reported here demonstrate that Se can inhibit the growth of *Foc* TR4 and increase the enzyme activities of the antioxidant system, including those of POD, PPO, and GSH-Px. Our findings are similar to the results of previous studies involving Se treatments. Se supplementation enhances the activities of antioxidant enzymes (POD, SOD, CAT, and PPO), thereby mitigating the accumulation of ROS and H_2_O_2_ in plants, ultimately reducing the disease incidence [29,44,56,57]. Appropriate Se supplementation can significantly enhance the activities of antioxidant enzymes in plants, thereby reducing the levels of ROS, potentially plants’ antioxidant defense mechanisms. The increase in antioxidant enzyme activity is an important response of banana against *Foc* TR4. The application of compound fungal biocontrol agents has been shown to induce systemic resistance in banana, characterized by increases in POD and CAT activity [58]. Other studies have shown that after being infected by the *Foc* TR4 pathogen, the levels of POD, CAT, and PAL activities, as well as those of oxidized glutathione and the genes encoding PPO (*PPO-1*, *PPO-2*, and *PPO-3*) significantly increase in resistant banana varieties, whereas there is no significant change in susceptible varieties [59,60]. Our results strongly support the hypothesis that Se could trigger the defense response by increasing those enzyme activities in banana against *Foc* TR4. Furthermore, Se could mitigate damage to the host by inhibiting the growth of fungi (Figure 2). Se has been shown to inhibit the growth of several fungal pathogens, including *F. oxysporum*, *F. graminearum*, *Botrytis cinerea*, *Aspergillus flavus*, and *Penicillium expansum* [29,35,40,61]. Additionally, it can prevent *F. graminearum* and *Alternaria solani* from expanding once they have invaded the host plant [32,62]. The reported inhibition of *Sclerotinia sclerotiorum* is currently the most convincing study. It was reported that Se inhibited the growth and germination of *S. sclerotiorum* mycelium, damaged the ultrastructure, diminished the capacity for acid production, lowered the activities of SOD and CAT, and elevated the levels of ROS and H_2_O_2_, collectively leading to a reduction in sclerotial formation in oilseed rape [27,31]. We also found that *Foc* TR4 growth was inhibited under Se concentrations greater than or equal to 10 mg/L and was completely killed under concentrations of 40 mg/L or higher. Collectively, our results demonstrate the Se distribution across various banana tissues and its induction of disease resistance by increasing the activities of antioxidant enzymes. However, the current understanding of the threshold at which Se inhibits plant growth and the underlying mechanisms remain unclear. Our next study will aim to elucidate the most effective forms, threshold ranges, and inhibitory mechanisms of Se in banana plants.

### 3.4. Applying Se in a Safe and Beneficial Way

The Se selected in this study is not only effectively in controlling banana *Foc* TR4 but also in benefiting human health through Se conversion in fruit. Se is widely but unevenly distributed in the natural environment, with TSe in soil significantly correlated with soil and climate conditions. The average TSe in global soil is 0.1–2.0 ppm, and Se-deficient areas are commonly found worldwide, with approximately 500 million to 1 billion people currently at risk of Se deficiency. In China, the median TSe in soil is 0.219 ppm, and most of the land is Se-deficient [15,63]. Se deficiency poses great health risks, leading to diseases such as Keshan disease, impaired fertility, and immune deficiency, in addition to causing food safety issues by decreasing As and Cd [57,64]. It is necessary to supplement Se in a scientifically reasonable manner to maintain human health [51]. Humans in Se-deficient areas rely on Se fertilizers to boost the Se levels in crops, thereby satisfying their dietary Se requirements [48,49,65]. Due to the myriad health benefits associated with Se-enriched fruits, there is a generally positive perception and recognition of these fruits for people. The prices of Se-enriched fruits are significantly higher than those of ordinary fruits, reflecting their popularity. However, there exist notable disparities in Se enrichment capacity among various fruits, with the collective enrichment potential generally being relatively modest [66,67]. Thus, Se-enriched banana has significant potential market value. When sodium selenate (Na_2_SeO_4_) and sodium selenite (Na_2_SeO_3_) were used to treat banana plantlets at different doses, it was found that Na_2_SeO_3_ was concentrated mainly in the roots and more prone to transfer to fruit [24]. The Se form we recommend to use is Na_2_SeO_3_, which is already included in the “List of Permitted Nutrient Fortifier Compound Sources” (GB14880-2012, [68]). Furthermore, the recommended effective dose for application to banana plantlets against *Foc* TR4 is very low (100–200 mg/plant). Our study revealed that the total Se content in fresh banana pulp was 39.6 ± 5.7 μg/kg and 43.2 ± 5.6 μg/kg after two field applications of Se (100 mg/plant and 200 mg/plant), meeting the Se-enrichment standard of 10~100 μg/kg (DB45/T1061-2014). This characteristic renders it challenging to attain the World Health Organization’s recommended daily adult intake of 400 μg [67]. Therefore, the effective form of Se^4+^ and its optimal dose, as demonstrated by our data, can alleviate the harm caused by *Foc* TR4, serving as an environmentally friendly control technology for soil-borne diseases, with profound significance for the sustainable development of the banana industry. At the same time, it effectively enriches Se in bananas, thereby benefiting human health.

This research outcome has a dual benefit: both as a Se-based means for controlling *Foc* TR4 and as a Se fortifier in plants. However, the dose effect is significant, and exceeding the recommended dose is not advisable [69]. Our research also indicates that surpassing a certain dose does not noticeably enhance banana *Foc* disease resistance. A Se dose of 400 mg/plant has an insignificant anti-disease effect, with a disease incidence of 85.0, and although fruits were formed, there was no harvest. Furthermore, this excessive application elevated Se accumulation in fruit to a level surpassing the standard (DB45/T1061-2014) of 100 μg/kg, which might pose a food safety risk. Previous studies by Zhang et al. (2018) [23] demonstrated that use of Se fertilizer significantly increased both banana yield and Se content, but the Se content in fresh fruit exceeded safe levels. It was recommended to reduce the Se fertilizer dose during planting, avoid spraying fruits directly, and minimize the pollution of fruit surfaces to enhance safety [23]. Soil applications of Na_2_SeO_4_ of 250 mg/plant and 500 mg/plant achieved the standard for Se-enriched banana, whereas the application of 750 mg/plant Na_2_SeO_4_ exceeded the upper limit of the Se-enriched standard [25]. This study confirmed that treatment of banana plants with 100–200 mg Se/plant after 5 months of growth can control *Foc* TR4 and yield Se -enriched bananas within safe limits. Future research will focus on optimizing the Se dose to effectively balance banana resistance to *Foc* TR4 with its Se enrichment effect. In the next step of our research, we will explore various approaches, such as controlling the Se content in plantlets, utilizing safe Se-containing organic substances, and inducing resistant metabolites—specifically lipids—produced by Se to enhance plant resistance.

## 4. Materials and Methods

### 4.1. Cultivation of Banana Plantlets After Se Application in the Greenhouse

Brazilian (*Musa* spp., AAA) plantlets were cultivated under various Se treatments in a greenhouse. Sodium selenite solid powder (Na_2_SeO_3_, Sigma-Aldrich, St. Louis, MO, USA) was selected as the form of Se^4+^ in this study. The greenhouse at Yunnan Agricultural University, located in Kunming, maintains a temperature range of 30 ± 5 °C. The humidity levels within the facility are consistently between 60% and 80%, while the average light intensity is approximately 80% that of natural light, ensuring optimal conditions for plant growth and research. The specific operational steps were carried out as follows: First, the Brazilian plantlets were transferred from culture bottles to nursing plates at an appropriate temperature (25–28 °C) and humidity (>90%) and cultured in a seedling tray (60 mm length × 60 mm width × 115 mm height) with wet coconut coir and matrix (volume ratio 1:1) for about 2 weeks. Then, surviving plantlets were transferred to plastic pots (diameter, 125 mm; height, 95 mm) containing natural soil and matrix (1:1 volume ratio) (weight of about 600 g and volume of about 0.7 L) for cultivation with the total Se content (TSe) (0.29 ± 0.02 ppm) in the soil and matrix. Cultivation continued for about 6 weeks until the plantlet length reached over 30 cm with 5–6 leaves. Then, the plantlets were used for Se treatments and/or *Foc* TR4 inoculation. Seven solutions of 14, 70, 140, 280, 560, 1120, and 2240 mg/L Se with water were prepared, and 50 mL of different Se solutions were poured into each plastic pot. Se treatments were applied, with final concentration gradients of 1, 5, 10, 20, 40, 80, and 160 mg/L. The plants were then grown for an additional period of time. A treatment using 50 mL of water was set as the control. In addition, we used flower-pot trays (diameter, 11.3 cm; height, 2.2 cm) to maintain the effective concentration of Se and increased the distance between treatment groups (20 cm) to prevent cross-contamination.

### 4.2. Measuring the Plant Height, Diameter of the Pseudostem, and Number of Leaves

Banana plantlets were assessed for plant height, diameter of the pseudostem, and the number of leaves 0 days, 7 days, 14 days, and 21 days post treatment with varying concentrations of Se. Plant height was measured using a flexible ruler to determine the vertical extent of the plant’s above-ground parts. At the base of the pseudostem, where it meets the soil, the diameter was measured using a caliper. The number of leaves represents the sum of leaves present on a plant’s above-ground parts. The experiment included 6 biological replicates per treatment. Statistical significance was determined between the treatment and the control using Student’s *t*-tests.

### 4.3. Validating the In Vitro Inhibition of Mycelial Foc TR4 Growth by Se

*Foc* TR4 used in this study is a highly pathogenic wild-type strain (15-1) isolated by our research group from Xishuangbanna, Yunnan province, China [70]. Each 100 mL of PDA medium was supplemented with 4 mL of a Se-diluted solution with ddH_2_O (25, 125, 250, 500, 1000, 2000, and 4000 mg/L), and PDA medium was supplemented with 4 mL of ddH_2_O as the control. After shaking thoroughly, the final concentrations of Se were as follows in the PDA medium: 1, 5, 10, 20, 40, 80, and 160 mg/L. Then, these solutions were poured into 6 culture dishes (diameter, 90 mm) for each concentration. *Foc* TR4 fungal blocks with 5 mm diameters were inoculated onto the middle of the PDA medium, and the colony diameter was measured after the fungi had been cultured for 1 to 6 days. Statistical significance was determined between the treatment and the control using Student’s *t*-tests. Photographs were taken to document the *Foc* TR4 culture on the seventh day. The experiment included 6 biological replicates per treatment. The *inhibition* ratios were calculated based on the colony diameter in the 6th day: *inhibition rate* (%) = (R_0_ − R_t_)/R_0_ × 100%, where R_t_ is the average *diameter* of the *treatment group* and R_0_ is the average diameter of the *control group*.

### 4.4. Disease Assessment and Sample Collection After Se Application in the Greenhouse

The purpose of this part was to evaluate the response of Brazilian (*Foc* TR4 susceptible) plantlets to *Foc* TR4 under different Se treatments in a greenhouse. The comprehensive design scheme of artificial inoculation is shown in Figure S3A(a,b). Banana plantlets treated with Se 14 days prior were used for *Foc* TR4 inoculation. The artificial injured-root inoculation method was used to inoculate plantlets with *Foc* TR4 according to Liu et al. (2021) [71]. Inoculated plantlets were then transferred to plastic pots (diameter, 125 mm; height, 95 mm) containing natural soil and matrix (1:1). The final concentration of the *Foc* TR4 inoculant was 2 × 10^6^ spores/g soil and matrix (1:1) for the plantlets treated with/without Se. The level of Fusarium wilt disease severity was assessed, and the disease index (DI) was calculated on the 42nd day post inoculation (dpi). Treatments were labeled as Control, Se01, Se05, Se10, Se20, Se40, Se80, and Se160 (Figure S3A(a)). Each treatment included 10 plantlets as biological replicates, and the experiments were repeated twice. The disease severity level was visually classified into categories ranging from 0 to 4, with 4 representing the most severe level according to the method proposed by Brake et al. (1995) and Xu et al. (2017) [72,73]. The criteria for the disease severity assessment are4 described as follows. Level 0: No streaking or yellowing of leaves. The plant appears healthy, with no discoloration of the corm region. Level 1: Slight streaking and yellowing of lower leaves, with 1–20% discoloration in the corm region. Level 2: Significant streaking and yellowing are observed in the lower leaves, with discoloration potentially beginning to affect younger leaves and 21–50% of the corm region discolored. Level 3: Extensive streaking and yellowing on most or all of the leaves, with more than 50% of the corm region discolored. Level 4: The entire corm region is discolored, and the plant is dead. The calculation formula is DI = Σ (the number of plants × surveyed disease severity level)/(total number of plants × 4) × 100 [74]. Student’s *t*-test was used to determine statistical differences between the two conditions, with significance determined at *p* < 0.05. Furthermore, root, leaf, and corm samples of banana plants under Se treatments (Se40, Se80) and the control were collected 7 dpi for TSe detection based on the DI results and plant growth.

Similarly, to evaluate the control effect of Se on *Foc* TR4, plantlets were inoculated first, then treated seven days later with 50 mL different concentrations of Se. The DI values calculated 42 dpi at final concentrations of 20, 40, 80, and 160 mg/L (Figure S3A(b)) according to the above method were labeled as Se20B, Se40B, Se80B, and Se160B, respectively. Each treatment included 10 plantlets as biological replicates, and the experiments were repeated twice.

### 4.5. Disease Assessment and Sample Collection After Se Application in the Field

In March 2022, Brazilian (Cavendish subgroup, AAA, *Foc* TR4 susceptible) plantlets were planted in Yuxi City (23.39° N, 101.94° E, 428 m), Yunnan Province, China. This field is characterized by a hot, dry valley climate, with an average annual temperature of 23.8 °C and precipitation of 800 to 1000 mm. The background value of the TSe in the plot was 0.12 ± 0.01 ppm. The experimental plantlets were managed under conventional banana plantation conditions within a single plot. At the base of each banana plant, a hole (radius of about 20 cm and depth of about 5 cm) was dug around the pseudostem for watering with Se solution and water as a control. During the banana seedling stage in July, two concentrations of sodium selenite solution with water (40 mg/L and 80 mg/L) were prepared, and 2.5 L of sodium selenite solution was used for drenching near the corm. The final doses of Se were 100 mg/plant and 200 mg/plant, and water only was used as the control. Each treatment consisted of 10 plants as biological replicates, labeled as Se100, Se200, and Control. The experiment was repeated twice. At the end of December, the mortality rate of plants during banana harvesting in the field was used as the basis for evaluating the effect of Se on *Foc* TR4. The evaluation method was performed according to Huang et al. (2005) [75]. During the banana harvest season, the disease grading standards were determined by the percentage of plant deaths caused by *Foc* TR4 and labeled as follows: high resistance, incidence rate ≤ 10%; resistance, incidence rate of 11–20%; moderate resistance, incidence rate of 21–40%; susceptibility, incidence rate of 41–60%; high susceptibility, incidence rate > 60%. Based on Student’s *t*-tests between two conditions, significant difference was determined at *p* < 0.05. In addition, tissues of each plant were collected for measurement of the TSe. The design scheme involving natural infection in the field and information about collected samples are shown in Figure S3B.

### 4.6. Changes in Antioxidant System Indicators of Banana Leaves After Se Application

The antioxidant system, crucial for eliminating ROS, was assessed through the activity of five key enzymes, including POD, PPO, SOD, CAT, and GSH-Px, as well as changes in GSH concentration, as per the reagent kit’s instructions. Yong leaves of banana plantlets treated with Se and *Foc* TR4 in greenhouse assessments were employed as the experimental materials. POD activity (ΔOD_470_/min/g) was measured by a microplate assay and a peroxidase assay kit (G0107W, Suzhou Greasy, Suzhou, China). CAT activity (μmol/L/min/mg) was determined by a visible colorimetric assay with a catalase kit (G0105W48, Suzhou Greasy, Suzhou, China). SOD activity (U/g) was quantified using the Water-Soluble Tetrazolium (WST-8) colorimetric method with a superoxide dismutase kit (G0101W, Suzhou Greasy, Suzhou, China). PPO activity (ΔOD_420_/min/g) was detected by a microplate assay with a polyphenol oxidase kit (G0113W, Suzhou Greasy, Suzhou, China). GSH-Px activity (U/mg pro) was determined by a colorimetric method using a glutathione peroxidase kit (A005-1-1, Nanjing Jiancheng, Nanjing, China). GSH concentration (μmol/L) was measured by a colorimetric method using a glutathione assay kit (A006-1-1, Nanjing Jiancheng, Nanjing, China). A standard curve was prepared according to the kit instructions for calculation of the GSH concentration. Each treatment consisted of 5 biological replicates. Student’s *t*-test was used to determine statistical significance between two conditions, with significant difference determined at *p* < 0.05.

### 4.7. Determination and Analysis of TSe in Banana Plants

According to the “National Food Safety Standard of the People’s Republic of China- Determination of Selenium in Food” (GB5009.93-2017 [76]), the TSe in samples were measured by hydride generation–atomic fluorescence spectrometry. The details are described as follows: (1) Sample collection: Each sample was cleaned with distilled water and dried, and 0.5 g to 3.0 g of the solid sample was weighed out. (2) Sample dissolution: Individual samples were added to 10 mL of a 9:1 HNO_3_-HClO_4_ mixture and subjected to cold digestion overnight. The mixture was then heated at 150 °C with 68% HNO_3_ until the solution became clear and colorless. The remaining volume should be approximately 2 mL. After cooling, 5 mL of 6 moL/L HCl solution was added, and the liquid continued heating until the solution become clear and colorless with white smoke. Then, 2.5 mL 100 g/L K₃[Fe(CN)₆] solution was added and diluted to 10 mL with water. (3) Measuring TSe: An atomic fluorescence spectrometer (AFS-9700A, Beijing Haiguang Instrument Co., Ltd., Beijing, China) was used to determine samples and standard curve samples with a 2 mL injection volume. (4) Calculation of TSe: TSe X (ppm) = (mass concentration of Se in the sample: ρ μg/L-mass concentration of Se in the blank: ρ0 μg/L) × total volume of sample digestion solution/(sample weight M g × 1000).

These experiments consisted of three biological replicates for the control, Se100, and Se200 treatments in the root, corm, leaf, pseudostem, and peduncle of banana plants and nine replicates of fruit collected in field. Additionally, three biological replicates each for the control, Se40, and Se80 treatments in the root, corm, and leaf of banana plantlets collected in a greenhouse, were also determined. Data are presented as the mean ± SE, and Student’s *t*-test was used to determine statistical significance between two conditions, with significant difference determined at *p* < 0.05.

## 5. Conclusions

This study has revealed, for the first time, that Se^4+^ not only significantly reduces the disease damage caused to banana by *Foc* TR4 by increasing the activity of antioxidant enzymes in the plant and inhibiting the growth of hyphae but also increases the diameter of the pseudostem and the number of leaves, enabling the harvest of Se-enriched bananas. The banana plants’ DI following *Foc* TR4 inoculation exhibited a strong negative correlation with TSe levels in the roots. The TSe levels in banana tissues were influenced by Se treatment rates. Exogenous field applications of Se^4+^ (100–200 mg per plant) are recommended to protect bananas from *Foc* TR4 and to produce safe and Se-enriched banana fruits. In summary, the Se^4+^ applied to banana plants in this research demonstrated dual benefits: reducing Fusarium wilt disease incidence and improving fruit quality. These findings support a novel approach to control soil-borne diseases in agriculture, the mechanism of which will be elucidated in further research.

## Figures and Tables

**Table 1:** Changes in TR4 community diameter under different concentrations of Se^4+^ treatment.

Se^4+^ Concentration *	Diameter After 1 Day (mm)	Diameter After 2 Day (mm)	Diameter After 3 Day (mm)	Diameter After 4 Day (mm)	Diameter After 5 Day (mm)	Diameter After 6 Day (mm)
Control	9.0 ± 2.4	25.5 ± 4.3	40.4 ± 4.7	55.9 ± 5.3	69.5 ± 4.3	76.9 ± 2.3
1 mg/L	8.9 ± 0.7	24.2 ± 1.1	39.8 ± 1.4	52.6 ± 2.0	68.4 ± 3.0	79.1 ± 0.7
5 mg/L	8.4 ± 0.6	22.4 ± 1.8	36.5 ± 1.9	49.3 ± 2.2 **	63.9 ± 3.5 *	77.3 ± 1.6
10 mg/L	7.9 ± 0.7	23.8 ± 2.2	37.1 ± 2.4	50.2 ± 2.4 **	62.0 ± 2.8 ***	70.6 ± 3.3 **
20 mg/L	5.7 ± 1.6 **	20.8 ± 2.0 *	32.4 ± 6.5 *	44.6 ± 6.0 **	56.5 ± 6.4 ***	64.0 ± 6.1 ***
40 mg/L	1.8 ± 0.8 ***	3.9 ± 1.9 ***	3.2 ± 1.8 ***	4.5 ± 2.0 ***	5.0 ± 2.6 ***	5.4 ± 2.9 ***
80 mg/L	1.6 ± 0.5 ***	2.8 ± 1.1 ***	2.1 ± 0.8 ***	2.6 ± 0.8 ***	2.1 ± 1.2 ***	2.2 ± 1.2 ***
160 mg/L	1.4 ± 0.5 ***	2.3 ± 1.0 ***	1.8 ± 0.7 ***	1.6 ± 0.5 ***	1.7 ± 0.4 ***	1.8 ± 0.3 ***

The data were presented as the mean ± SE, with *n* = 6 replicates. Statistical significance was determined between the treatment and the control using Student’s *t*-tests, with * *p* < 0.05, ** *p* < 0.01, and *** *p* < 0.001.

**Table 2:** TSe levels in banana tissues at the fruit-setting stage in the field after Se100 and Se200 applications.

Tissue\ *	Control (μg/kg)	Se100 (μg/kg)	Se200 (μg/kg)
Root	330.0 ± 150.8 cA	1337.7 ± 88.9 bA	2891.0 ± 770.9 aA
Corm	21.3 ± 2.5 cB	71.3 ± 10.7 bC	185.3 ± 56.1 aC
Pseudostem	67.3 ± 24.5 bB	338.3 ± 88.8 aB	335.3 ± 54.2 aBC
Leaves	42.7 ± 23.4 cB	128.7 ± 28.2 bC	302.0 ± 37.3 aC
Peduncle	35.3 ± 1.7 bB	347.3 ± 23.6 aB	488.7 ± 321.1 aBC

* Data are presented as the mean ± SE, with *n* = 3 replicates. Student’s *t*-test was used to determine statistical significance between two conditions, and the significant difference was determined at *p* < 0.05. The results were analyzed using labels a–c to compare treatment effects on one tissue, while A–C represent comparisons among different tissues under the same treatment.

## Data Availability

The data presented in this study are available in this article.

## Supplementary Materials

The following supporting information can be downloaded at https://www.mdpi.com/article/10.3390/plants13233435/s1: Figure S1. The variation in the disease severity level of banana plants treated with Se after being inoculated with Foc TR4 in greenhouse experiments. Figure S2. Severe occurrence and pseudostem symptoms of Foc TR4 disease during the fruiting period in the field. Figure S3. The comprehensive sample design scheme involving artificial inoculation in a greenhouse (A) and natural infection in the field (B).

## References

[1] Bubici G., Kaushal M., Prigigallo M.I., Cabanás C.G.L., Mercado-Blanco J. (2019). Biological control agents against Fusarium wilt of banana. Front. Microbiol..

[2] Bai T., Qin M., Li X., Fan H., Xu S., Zeng L., Zheng S. (2020). An additional threat to ‘Cavendish’ banana growers and traders: The infection of banana peduncles by *Fusarium oxysporum* f. sp. *cubense* tropical race 4 (*Foc* TR4). Plant Health Prog..

[3] García-Bastidas F., Ordóñez N., Konkol J., Al-Qasim M., Naser Z., Abdelwali M., Salem N., Waalwijk C., Ploetz R.C., Kema G.H.J. (2014). First report of *Fusarium oxysporum* f. sp. *cubense* tropical race 4 associated with Panama disease of banana outside Southeast Asia. Plant Dis..

[4] Chittarath K., Mostert D., Crew K., Viljoen A., Thomas J. (2017). First report of *Fusarium oxysporum* f. sp. *cubense* tropical race 4 (vcg 01213/16) associated with Cavendish bananas in Laos. Plant Dis..

[5] Dita M., Barquero M., Heck D., Mizubuti E.S.G., Staver C.P. (2018). Fusarium wilt of banana: Current knowledge on epidemiology and research needs toward sustainable disease management. Front. Plant Sci..

[6] Zheng S., García-Bastidas F.A., Li X., Zeng L., Bai T., Xu S., Yin K., Li H., Fu G., Yu Y. (2018). New geographical insights of the latest expansion of *Fusarium oxysporum* f. sp. *cubense* tropical race 4 into the Greater Mekong Subregion. Front. Plant Sci..

[7] Viljoen A., Mostert D., Chiconela T., Beukes I., Molina A.B. (2020). Occurrence and spread of the banana fungus *Fusarium oxysporum* f. sp. *cubense* TR4 in Mozambique. S. Afr. J. Sci..

[8] Acuña R., Rouard M., Leiva A., Marques C., Olortegui J., Ureta C., Cabrera-Pintado R., Rojas J., Lopez-Alvarez D., Cenci A. (2022). First report of *Fusarium oxysporum* f. sp. *cubense* tropical race 4 causing Fusarium wilt in Cavendish bananas in Peru. Plant Dis..

[9] Mmadi M., Azali H.A., Mostert D., Robene I., Viljoen A. (2023). First report of Fusarium wilt of Cavendish bananas caused by *Fusarium oxysporum* f. sp. *cubense* tropical race 4 in the Grande Comoros island. Plant Dis..

[10] Heslop-Harrison J., Schwarzacher T. (2007). Domestication, genomics and the future for banana. Ann. Bot..

[11] Ploetz R.C. (2015). Management of Fusarium wilt of banana: A review with special reference to tropical race 4. Crop Prot..

[12] Li H., Li Y., Nie Y. (2019). Research status of occurrence and control of Fusarium wilt of banana. J. South China Agric. Univ..

[13] Schwarz K., Foltz C.M. (1957). Selenium as an integral part of factor 3 against dietary necrotic liver degeneration. J. Am. Chem. Soc..

[14] Rotruck J.T., Pope A.L., Ganther H.E., Swanson A.B., Hafeman D.G., Hoekstra W.G. (1973). Selenium: Biochemical role as a component of glutathione peroxidase. Science.

[15] Wang Q.Q., Yu S.C., Xu C.D., Liu J.J., Li Y.Q., Zhang M.H., Long X.J., Liu Y.N., Bi Y.F., Zhao W.H. (2020). Association between selenium in soil and diabetes in Chinese residents aged 35–74 years: Results from the 2010 national survey of chronic diseases and behavioral risk factors surveillance. Biomed. Environ. Sci..

[16] Zhang J., Taylor W.E., Bennett K., Saad R., Rayman M.P. (2020). Association between regional selenium status and reported outcome of COVID-19 cases in China. Am. J. Clin. Nutr..

[17] Hossain A., Skalicky M., Brestic M., Maitra S., Sarkar S., Ahmad Z., Vemuri H., Garai S., Mondal M., Bhatt R. (2021). Selenium biofortification: Roles, mechanisms, responses and prospects. Molecules.

[18] Broadley M.R., Alcock J., Alford J., Cartwright P., Foot I., Fairweather-Tait S.J., Hart D.J., Hurst R., Knott P., McGrath S.P. (2010). Selenium biofortification of high-yielding winter wheat (*Triticum aestivum* L.) by liquid or granular se fertilisation. Plant Soil.

[19] Feng T., Chen S.S., Gao D.Q., Liu G.Q., Bai H.X., Li A., Peng L.X., Ren Z.Y. (2015). Selenium improves photosynthesis and protects photosystem II in pear (*Pyrus bretschneideri*), grape (*Vitis vinifera*), and peach (*Prunus persica*). Photosynth. Int. J. Photosynth. Res..

[20] Xia Q., Yang Z., Shui Y., Liu X., Chen J., Khan S., Wang J., Gao Z. (2020). Methods of selenium application differentially modulate plant growth, selenium accumulation and speciation, protein, anthocyanins and concentrations of mineral elements in purple-grained wheat. Front. Plant Sci..

[21] Li D., Zhou C., Zhang J., An Q., Wu Y., Li J., Pan C. (2020). Nanoselenium foliar applications enhance the nutrient quality of pepper by activating the capsaicinoid synthetic pathway. J. Agric. Food Chem..

[22] Li D., Zhou C., Zou N., Wu Y., Pan C. (2021). Nanoselenium foliar application enhances biosynthesis of tea leaves in metabolic cycles and associated responsive pathways. Environ. Pollut..

[23] Zhang Y.F., Qin Y.H., He M.J., Su X.J., Huang W.J., Wei X.Y. (2018). Preliminary study on the effect of yield and selenium content of banana by foliar spraying with different selenium fertilizers. J. Guangxi Agric..

[24] Liu J., Wu Y., Mou H., Peng J., Tian Q., Lei X., Wei S., Zhang Y., Huang S., Huang W. (2021). Effects of selenium application at the seedling stage on plant growth and fruit quality of banana. Chin. J. Trop. Agric..

[25] Liu J., Tian Q., Huang W., Wu Y., Peng J., Zhang Y., Xie R., Wei S., Mou H., Wei D. (2022). Effects of selenium application on the plant growth, physiology and fruit quality of three varieties of banana. Guihaia.

[26] Hanson B., Garifullina G.F., Lindblom S.D., Wangeline A., Ackley A., Kramer K., Norton A.P., Lawrence C.B., Pilon-Smits E.A.H. (2003). Selenium accumulation protects *Brassica juncea* from invertebrate herbivory and fungal infection. New Phytol..

[27] Liu K., Cai M., Hu C., Sun X., Cheng Q., Jia W., Yang T., Nie M., Zhao X. (2019). Selenium (se) reduces sclerotinia stem rot disease incidence of oilseed rape by increasing plant se concentration and shifting soil microbial community and functional profiles. Environ. Pollut..

[28] Cheng Q., Jia W., Hu C., Shi G., Yang D., Cai M., Zhan T., Tang Y., Zhou Y., Sun X. (2020). Enhancement and improvement of selenium in soil to the resistance of rape stem against *Sclerotinia sclerotiorum* and the inhibition of dissolved organic matter derived from rape straw on mycelium. Environ. Pollut..

[29] Li Q., Xian L., Yuan L., Lin Z., Chen X., Wang J., Li T. (2023). The use of selenium for controlling plant fungal diseases and insect pests. Front. Plant Sci..

[30] Wu Z., Yin X., Banuelos G.S., Lin Z.Q., Zhu Z., Liu Y., Yuan L., Li M. (2016). Effect of selenium on control of postharvest gray mold of tomato fruit and the possible mechanisms involved. Front. Microbiol..

[31] Cheng Q., Hu C., Jia W., Cai M., Zhao Y., Tang Y., Yang D., Zhou Y., Sun X., Zhao X. (2019). Selenium reduces the pathogenicity of *Sclerotinia sclerotiorum* by inhibiting sclerotial formation and germination. Ecotoxicol. Environ. Saf..

[32] Mao X., Hua C., Yang L., Zhang Y., Li T. (2020). The effects of selenium on wheat Fusarium head blight and don accumulation were selenium compound-dependent. Toxins.

[33] Mao X., Li P., Li T., Zhao M., Yu L. (2020). Inhibition of mycotoxin deoxynivalenol generation by using selenized glucose. Chin. Chem. Lett..

[34] Li Z.Y., Guo S.Y., Li L. (2003). Bioeffects of selenite on the growth of *Spirulina platensis* and its biotransformation. Bioresour. Technol..

[35] Wu Z.L., Yin X.B., Lin Z.Q., Banuelos G.S., Yuan L.X., Liu Y., Li M. (2014). Inhibitory effect of selenium against *Penicillium expansum* and its possible mechanisms of action. Curr. Microbiol..

[36] Xu J., Jia W., Hu C., Nie M., Ming J., Cheng Q., Cai M., Sun X., Li X., Zheng X. (2020). Selenium as a potential fungicide could protect oilseed rape leaves from *Sclerotinia sclerotiorum* infection. Environ. Pollut..

[37] Bollina V., Kumaraswamy G.K., Kushalappa A.C., Choo T.M., Dion Y., Rioux S., Faubert D., Hamzehzarghani H. (2010). Mass spectrometry-based metabolomics application to identify quantitative resistance-related metabolites in barley against Fusarium head blight. Mol. Plant Pathol..

[38] Rojas C.M., Senthil-Kumar M., Tzin V., Mysore K.S. (2014). Regulation of primary plant metabolism during plant-pathogen interactions and its contribution to plant defense. Front. Plant Sci..

[39] Zhao P., Liu L., Cao J., Wang Z., Zhao Y., Zhong N. (2022). Transcriptome analysis of tryptophan-induced resistance against potato common scab. Int. J. Mol. Sci..

[40] Companioni B., Medrano J., Torres J.A., Flores A., Rodriguez E., Benavides A. (2012). Protective action of sodium selenite against Fusarium wilt in tomato: Total protein contents, levels of phenolic compounds and changes in antioxidant potential. Acta Hortic..

[41] Zhu Z., Chen Y., Zhang X., Li M. (2016). Effect of foliar treatment of sodium selenate on postharvest decay and quality of tomato fruits. Sci. Hortic..

[42] Swarupa V., Ravishankar K.V., Rekha A. (2014). Plant defense response against *Fusarium oxysporum* and strategies to develop tolerant genotypes in banana. Planta.

[43] Li W., Qian C., Mo Y., Hu Y., Xie J. (2011). Tolerance of banana for Fusarium wilt is associated with early H_2_O_2_ accumulation in the roots. Afr. J. Biotechnol..

[44] Zang H., Ma J., Wu Z., Yuan L., Lin Z., Zhu R., Bañuelos G.S., Reiter R.J., Li M., Yin X. (2022). Synergistic effect of melatonin and selenium improves resistance to postharvest gray mold disease of tomato fruit. Front. Plant Sci..

[45] (2014). The Classification Criteria for Selenium Content in Selenium-Enriched Agricultural Products.

[46] Chen Z., Sun H., Hu T., Wang Z., Wu W., Liang Y., Guo Y. (2022). Sunflower resistance against *Sclerotinia sclerotiorum* is potentiated by selenium through regulation of redox homeostasis and hormones signaling pathways. Environ. Sci. Pollut. Res..

[47] Ikram M., Raja N.I., Mashwani Z.-U.-R., Omar A.A., Mohamed A.H., Satti S.H., Zohra E. (2022). Phytogenic selenium nanoparticles elicited the physiological, biochemical, and antioxidant defense system amelioration of huanglongbing-infected ‘kinnow’ mandarin plants. Nanomaterials.

[48] Zhou X., Yang J., Kronzucker H.J., Shi W. (2020). Selenium biofortification and interaction with other elements in plants: A review. Front. Plant Sci..

[49] Trippe-III R.C., Pilon-Smits E.A.H. (2021). Selenium transport and metabolism in plants: Phytoremediation and biofortification implications. J. Hazard. Mater..

[50] Hawrylak-Nowak B., Matraszek R., Pogorzelec M. (2015). The dual effects of two inorganic selenium forms on the growth, selected physiological parameters and macronutrients accumulation in cucumber plants. Physiol. Plant..

[51] Chao W., Rao S., Chen Q., Zhang W., Liao Y., Ye J., Cheng S., Yang X., Xu F. (2022). Advances in research on the involvement of selenium in regulating plant ecosystems. Plants.

[52] Li H.F., McGrath S.P., Zhao F.J. (2008). Selenium uptake, translocation and speciation in wheat supplied with selenate or selenite. New Phytol..

[53] Wang P., Menzies N.W., Lombi E., McKenna B.A., James S., Tang C., Kopittke P.M. (2015). Synchrotron-based x-ray absorption near-edge spectroscopy imaging for laterally resolved speciation of selenium in fresh roots and leaves of wheat and rice. J. Exp. Bot..

[54] Huang Q.Q., Wang Q., Wan Y.N., Yu Y., Jiang R.F., Li H.F. (2017). Application of X-ray absorption near edge spectroscopy to the study of the effect of sulphur on selenium uptake and assimilation in wheat seedlings. Biol. Plant..

[55] White P.J. (2018). Selenium metabolism in plants. Biochim. Biophys. Acta (BBA)-Gen. Subj..

[56] Shi M.T., Zhang T.J., Fang Y., Pan C.P., Fu H.Y., Gao S.J., Wang J.D. (2023). Nano-selenium enhances sugarcane resistance to *Xanthomonas albilineans* infection and improvement of juice quality. Ecotoxicol. Environ. Saf..

[57] Chen X., Jiang Y., Wang C., Yue L., Li X., Cao X., White J.C., Wang Z., Xing B. (2024). Selenium nanomaterials enhance sheath blight resistance and nutritional quality of rice: Mechanisms of action and human health benefit. ACS Nano.

[58] Gui S. (2015). Effectiveness and Mechanism of Control of Fusarium Wilt Disease of Banana by Application of Alkaline Fertilizer and Biocontrol Agents. Ph.D. Dissertation.

[59] Li W., Li C., Sun J., Peng M. (2017). Metabolomic, biochemical, and gene expression analyses reveal the underlying responses of resistant and susceptible banana species during early infection with *Fusarium oxysporum* f. sp. *cubense*. Plant Dis..

[60] Wei D., Wei L., Zhou W., Qin L., Huang S., Tian D., Li C., Long S., He Z., Wei S. (2021). Effects of *Fusarium oxysporum* f. sp. *cubense* on antioxidant capacity in roots of different resistant banana varieties. J. South. Agric..

[61] Zohri A.A., Saber S.M., Mostafa M.E. (1997). Effect of selenite and tellurite on the morphological growth and toxin production of *Aspergillus parasiticus* var. Globosus imi 120920. Mycopathologia.

[62] Joshi S.M., Britto S.D., Jogaiah S., Ito S.I. (2019). Mycogenic selenium nanoparticles as potential new generation broad spectrum antifungal molecules. Biomolecules.

[63] Winkel L.H.E., Johnson A., Lenz M., Grundl T., Leupin O.X., Amini M., Charlet L. (2012). Environmental selenium research: From microscopic processes to global understanding. Environ. Sci. Technol..

[64] Zhang X., Chen L., Leng R., Zhang J., Zhou Y., Zhang Y., Yang S., He K., Huang B. (2020). Mechanism study of the beneficial effect of sodium selenite on metabolic disorders in imidacloprid-treated garlic plants. Ecotoxicol. Environ. Saf..

[65] Kan X., Hu P., Chen B. (2021). Effects of exogenous selenium fertilizer on crop growth, quality and nutrient element content. Fertil. Health.

[66] Sun X., Ling M., Luo Y., Kou L. (2023). Development status and countermeasures of selenium-rich fruits in chongqing region. South China Fruits.

[67] Qin Y., Huang C., Huang G., Li H., Shohag M.J.I., Gu M., Shen F., Lu D., Zhang M., Wei Y. (2023). Relative bioavailability of selenium in rice using a rat model and its application to human health risk assessment. Environ. Pollut..

[68] (2012). Food Nutritional Fortifier Usage Standards.

[69] Jiang X.L., Qiao Y.T., Li X.J., Wang L., Xue Y.H., Xia H.Y. (2021). Effects of foliar spraying of excessive selenium on yields and contents of selenium and mineral elements of maize. J. Nucl. Agric. Sci..

[70] Zhang L., Yuan T., Wang Y., Zhang D., Zheng S. (2018). Identification and evaluation of resistance to *Fusarium oxysporum* f. sp. *cubense* tropical race 4 in *Musa acuminata* Pahang. Euphytica.

[71] Liu L.N., Yang B.M., Wang Y.F., Zeng L., Huang Y.L., Li Y.P., Yin K.S., Li X.D., Peng X.B., Xu S.T. (2021). Assessing of imported banana germplasms resistance to *Fusarium oxysporum* f. sp. *cubense* tropical race 4. South China Fruits.

[72] Brake V.M., Pegg K.G., Irwin J.A.G., Chaseling J. (1995). The influence of temperature, inoculum level and race of *Fusarium oxysporum* f. sp. *cubense* on the disease reaction of banana cv. Cavendish. Aust. J. Agric. Res..

[73] Xu S.T., Bai T.T., Zhang L., Fan H.C., Yang P.W., Yin K.S., Zeng L., Li X.D., Guo Z.X., Yang B.M. (2017). Evaluation of different banana varieties on Fusarium wilt TR4 resistance by phenotypic symptom and real-time quantitative PCR. Southwest China J. Agric. Sci..

[74] Mckinney H.H. (1923). Influence of soil temperature and moisture on infection of wheat seedlings by *Helmintosporium sativum*. J. Agric. Res..

[75] Huang B., Xu L., Yang H., Tang X., Wei Y., Qiu J., Li G. (2005). Preliminary results of field evaluation of banana germplasm resistant to Fusarium wilt disease. Guangdong Agric. Sci..

[76] (2017). National Food Safety Standard—Determination of Selenium in Foods..

