# Supplementary material for: The Dual Effect of Selenium Application in Reducing Fusarium Wilt Disease Incidence in Banana and Producing Se-Enriched Fruits
Source: Plants (Basel). 2024 Dec 6;13(23):3435. doi: 10.3390/plants13233435 (PMC11644460; doi:10.3390/plants13233435)
Supplement: Supplementary file 1 [file plants-13-03435-s001.zip › plants-3323872-supplementary.pptx]

## Slide 1
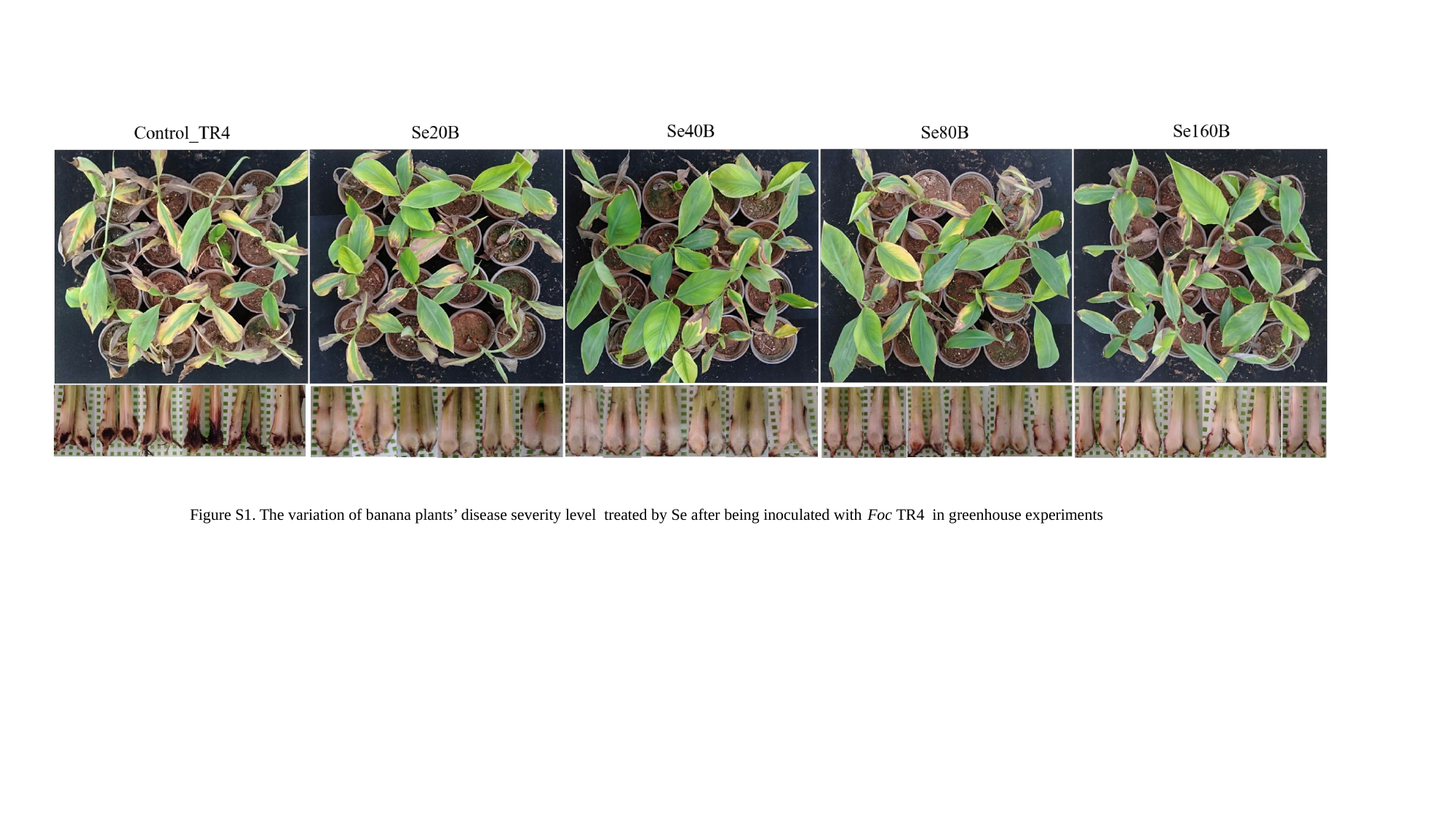

Figure S1. The variation of banana plants’ disease severity level treated by Se after being inoculated with Foc TR4 in greenhouse experiments

## Slide 2
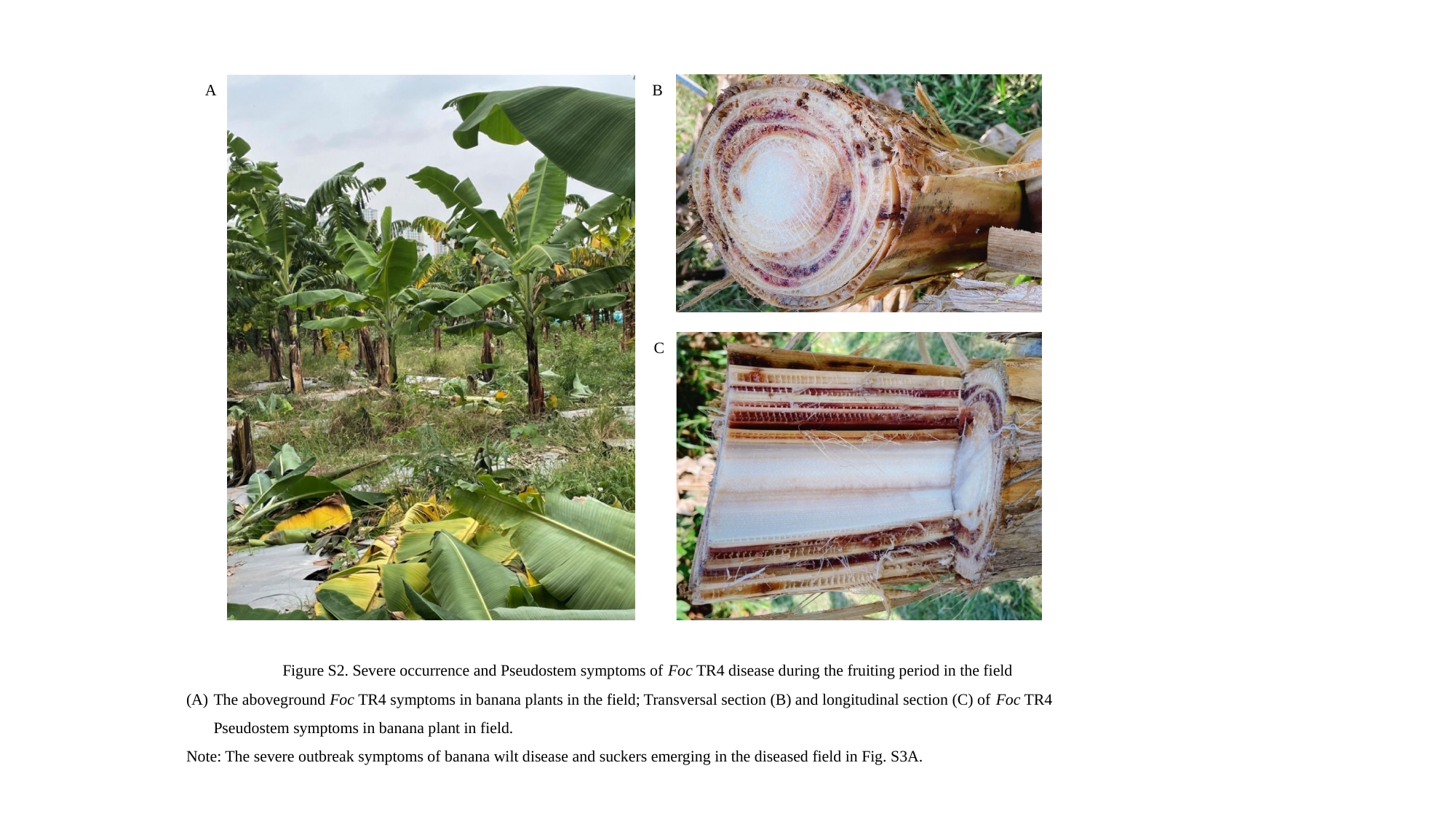

A
B
C
Figure S2. Severe occurrence and Pseudostem symptoms of Foc TR4 disease during the fruiting period in the field
The aboveground Foc TR4 symptoms in banana plants in the field; Transversal section (B) and longitudinal section (C) of Foc TR4 Pseudostem symptoms in banana plant in field.
Note: The severe outbreak symptoms of banana wilt disease and suckers emerging in the diseased field in Fig. S3A.

## Slide 3
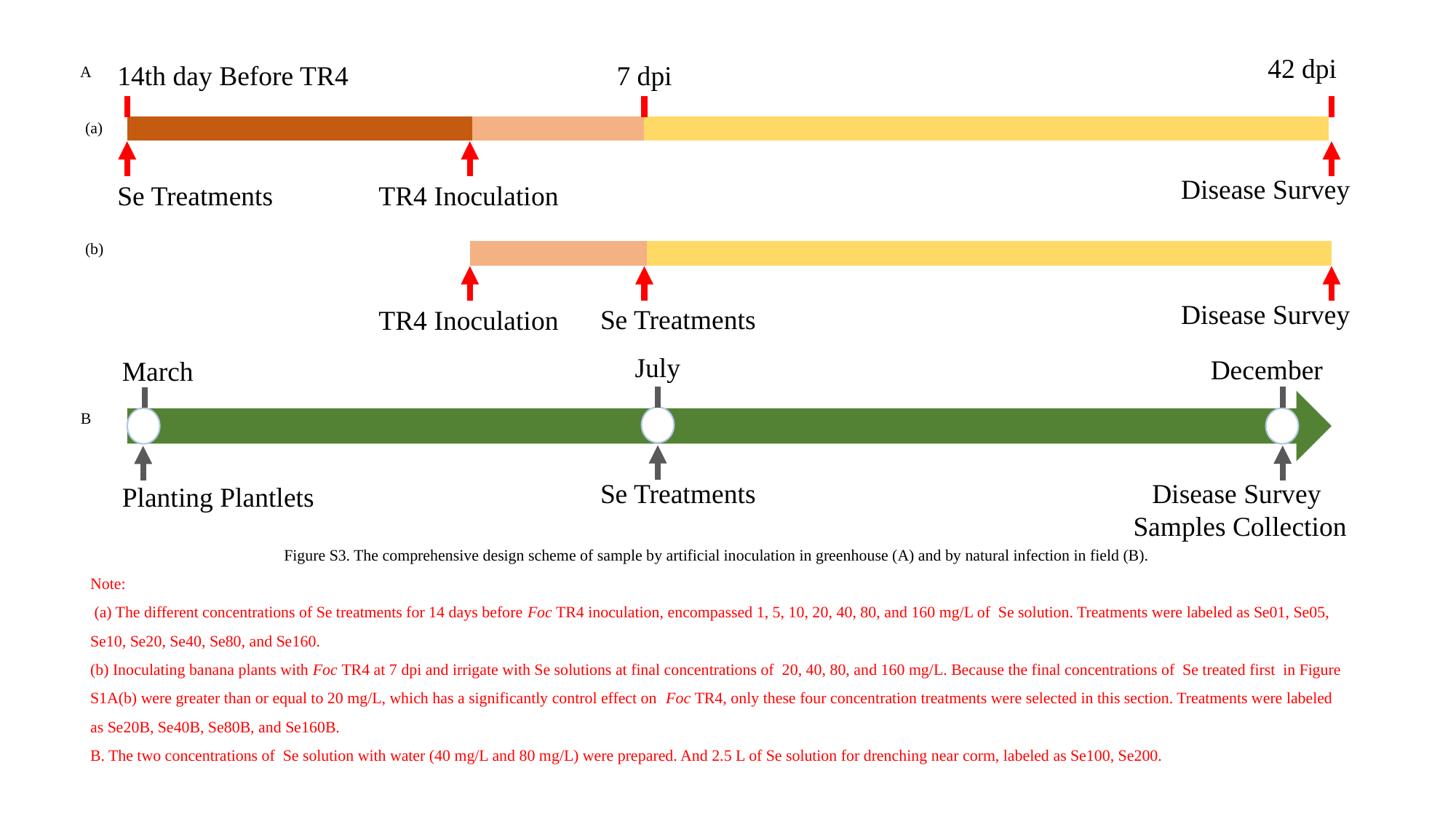

42 dpi
14th day Before TR4
7 dpi
Disease Survey
Se Treatments
TR4 Inoculation
A
Disease Survey
Se Treatments
TR4 Inoculation
July
December
March
B
Se Treatments
Disease Survey
Samples Collection
Planting Plantlets
(a)
(b)
Figure S3. The comprehensive design scheme of sample by artificial inoculation in greenhouse (A) and by natural infection in field (B).
Note:
 (a) The different concentrations of Se treatments for 14 days before Foc TR4 inoculation, encompassed 1, 5, 10, 20, 40, 80, and 160 mg/L of Se solution. Treatments were labeled as Se01, Se05, Se10, Se20, Se40, Se80, and Se160.
(b) Inoculating banana plants with Foc TR4 at 7 dpi and irrigate with Se solutions at final concentrations of 20, 40, 80, and 160 mg/L. Because the final concentrations of Se treated first in Figure S1A(b) were greater than or equal to 20 mg/L, which has a significantly control effect on Foc TR4, only these four concentration treatments were selected in this section. Treatments were labeled as Se20B, Se40B, Se80B, and Se160B.
B. The two concentrations of Se solution with water (40 mg/L and 80 mg/L) were prepared. And 2.5 L of Se solution for drenching near corm, labeled as Se100, Se200.
